# Anterior segment optical coherence tomography enables repeat Descemet Membrane Endothelial Keratoplasty instead of penetrating keratoplasty in a patient with a prominent corneal opacity following DMEK failure: a case report

**DOI:** 10.1186/s12886-026-05070-7

**Published:** 2026-07-13

**Authors:** Verena A. Englmaier, Stefanie Bobe, Nina Rolf, Matthias F. Kriegel, Peter Barth, Nicole Eter, Lamis Baydoun

**Affiliations:** 1 Department of Ophthalmology, University Medical Center Münster, Münster, Germany; 2 Gerhard-Domagk-Institute of Pathology, University Hospital Münster, Münster, Germany; 3 ELZA Institute, Dietikon/Zurich, Switzerland

**Keywords:** Descemet membrane endothelial keratoplasty, Endothelial failure, Corneal scar, Penetrating keratoplasty, Slit-lamp biomicroscopy, Anterior segment optical coherence tomography, Corneal vascularization, Systemic immunosuppression

## Abstract

**Background:**

Persistent corneal edema in eyes with endothelial dysfunction, such as graft failure, may lead to severe corneal opacification obscuring deeper ocular structures. In such cases, penetrating keratoplasty (PKP) has traditionally been preferred over posterior lamellar keratoplasty techniques to remove the edematous and opaque corneal tissue and replace it with a clear full-thickness graft.

**Case presentation:**

A 48-year-old woman was referred for PKP one year following primary Descemet Membrane Endothelial Keratoplasty (DMEK) failure in the left eye. As slit-lamp biomicroscopy showed progressive corneal edema with marked corneal opacification, the patient was scheduled for PKP. However, on the day of surgery, an anterior segment optical coherence tomography (AS-OCT) revealed a dense subepithelial hyperreflective layer anterior to the hypodense corneal stroma, corresponding to the opacified tissue. Given the suspected interface between the two layers, the surgical strategy was changed to anterior lamellar keratectomy combined with repeat DMEK. Intraoperatively, the fibrotic tissue could be separated from the underlying, relatively clear cornea so that DMEK could be performed in the same session. Six weeks postoperatively, best-corrected visual acuity (BCVA) had improved from counting fingers to 20/40 (0.5), and AS-OCT-based pachymetry had decreased from 1200 µm to 470 µm.

**Conclusion:**

Long-standing edema due to endothelial dysfunction may induce advanced corneal opacification, implying that PKP may be required to fully restore vision. Preoperative AS-OCT may be useful in such cases to distinguish separable subepithelial fibrosis from stromal involvement. Hereby the less invasive DMEK procedure may be performed while avoiding full-thickness transplantation with its associated complications.

## Background

Descemet membrane endothelial keratoplasty (DMEK) has become the standard of care for eyes with corneal edema due to endothelial dysfunction [1].

Long-standing edema can lead to corneal fibrosis that may negatively impact postoperative visual performance [2, 3]. However, DMEK can still provide acceptable outcomes in cases with preexisting mild corneal scarring secondary to chronic edema [4]. In eyes with Fuchs endothelial corneal dystrophy (FECD) and mild scarring, early treatment is often recommended to benefit postoperative visual acuity [5]. In contrast, in significantly opacified corneas, DMEK may not always be feasible and the degree of stromal involvement is often unclear. Therefore, penetrating keratoplasty (PKP) is usually recommended to excise the entire (partially) scarred corneal layers and replace them with a clear full-thickness transplant [3].

While ´low-risk´ PKP has a promising prognosis when performed for certain underlying diseases such as keratoconus, the risk of graft rejection and subsequent failure increases in the presence of corneal neovasculatization as the placement of the donor cornea button within an inflammatory, vascularized host bed will facilitate immune cells to reach and reject the graft [6, 7]. Systemic immunosuppressants, such as cyclosporine may prevent rejection but entail undesirable systemic adverse reactions [8].

We report a 48-year-old woman that was referred for PKP and systemic immunosuppression due to a dense corneal fibrosis with vascularizations following primary DMEK failure, who was successfully treated with lamellar keratectomy and repeat DMEK while avoiding full-thickness transplantation.

## Case presentation

**Fig. 1:**
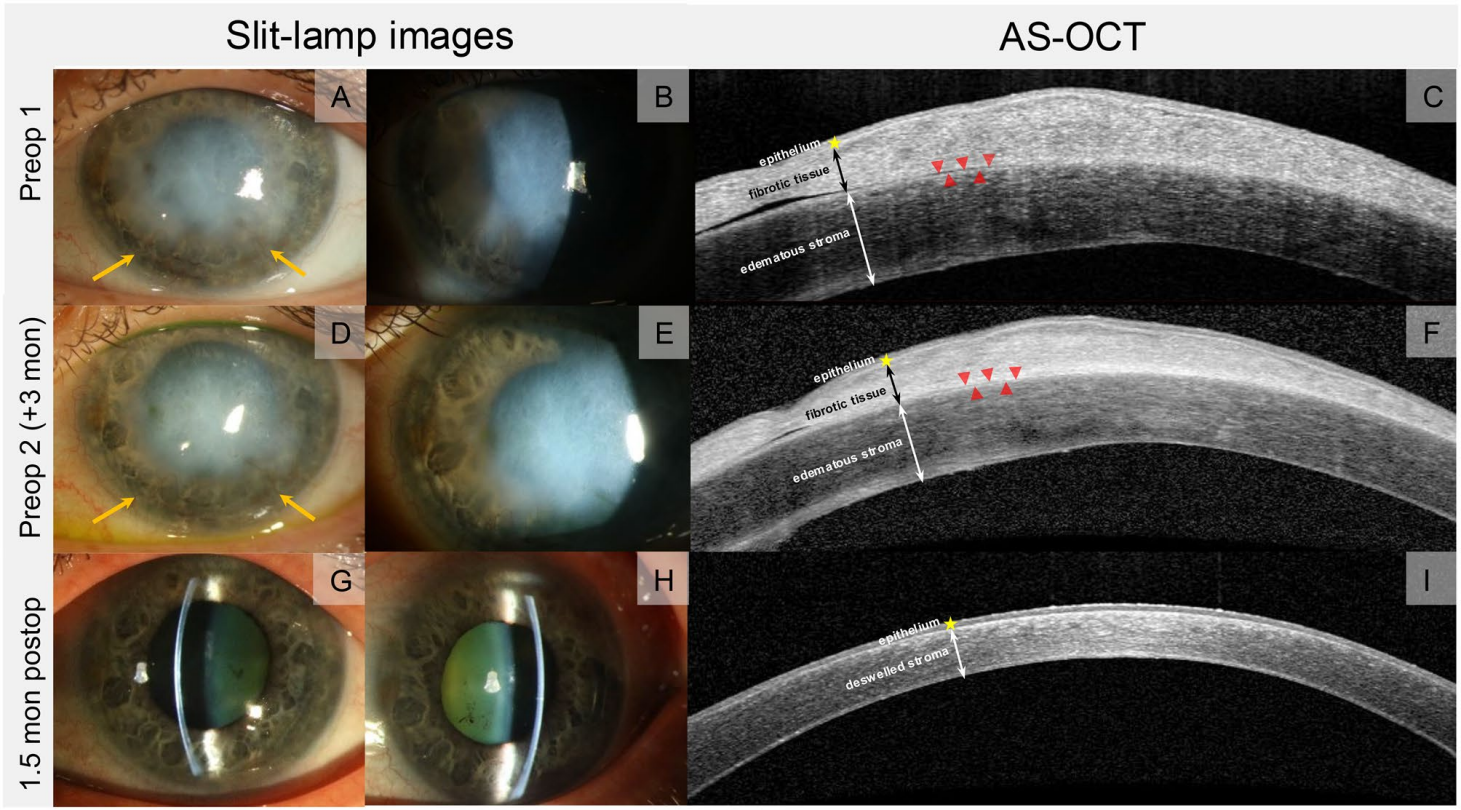
Preoperative and postoperative slit-lamp and anterior segment-optical coherence tomography (AS-OCT) images. Upper row; initial presentation showing a dense corneal opacification on slit-lamp biomicroscopy (**A, B**) and some corneal vascularizations (A, orange arrows). On AS-OCT (**C**), The opacification was hyperdense (black double-headed arrow) and clearly delineated from the overlying epithelium (star) and the underlying hypodense stroma (white double-headed arrow) creating an interface (red arrowheads) between both layers. The opacification measured 660 µm and the entire cornea approximately 1200 µm. Middle row; three months later and one day before repeat Descemet membrane endothelial keratoplasty (DMEK), the opacification had progressed slightly as seen on the slit-lamp images (**D, E**); The vascularizations were stable (orange arrows). On AS-OCT (**F**), The subepithelial layer appeared slighty more hyperdense (black double-headed arrow). The thickness of the subepithelial layer and entire cornea was stable. The interface between the hyperdense layer (black double-headed arrow) and the hypodense stroma (white double-headed arrow) appeared as a darker line (red arrowheads). Lower row; postoperative slit-lamp images 1.5 months after DMEK show a relatively clear cornea and a moderate cataract (**G, H**). AS-OCT showed a thinner cornea of 470 µm with no recurrence of the hyperdense layer

A 48-year-old woman was referred to our cornea clinic for PKP after failed primary phakic DMEK for FECD in her left eye (LE) one year earlier. Best-corrected visual acuity (BCVA) was 20/30 (0.63) in her right eye (RE) and counting fingers (CF) in her LE. Slit-lamp biomicroscopy revealed a prominent corneal scar with embedded neovascularization (Fig. 1). Therefore, systemic immunosuppressive treatment combined with PKP was suggested to improve visual acuity and reduce the risk of graft rejection. However, the patient suffered from multiple systemic diseases (among others obesity, type 1 diabetes) which increased the risks associated with systemic immunosuppression.

When the patient then presented three months later for surgery, the opacification appeared denser, further impairing proper visualization of the anterior chamber (Fig. 1). Anterior segment optical coherence tomography (AS-OCT) images were then assessed for the first time and revealed a thick, compact well-defined hyperreflective layer located between the hyporeflective corneal epithelium and the underlying stroma that most likely corresponded to the whitish opacity seen on slit-lamp examination (Fig. 1). AS-OCT-based pachymetry (full corneal thickness including the hyperreflective layer) measured approximately 1200 µm. Because a clear cleavage plane was visible between the hyperreflective suepithelial layer and the intact anterior stroma, and the stroma resembled that of an edematous cornea without hyperreflective features suggestive of scarring (Fig. 1), we decided to perform repeat DMEK combined with lamellar keratectomy instead of PKP.

**Fig. 2:**
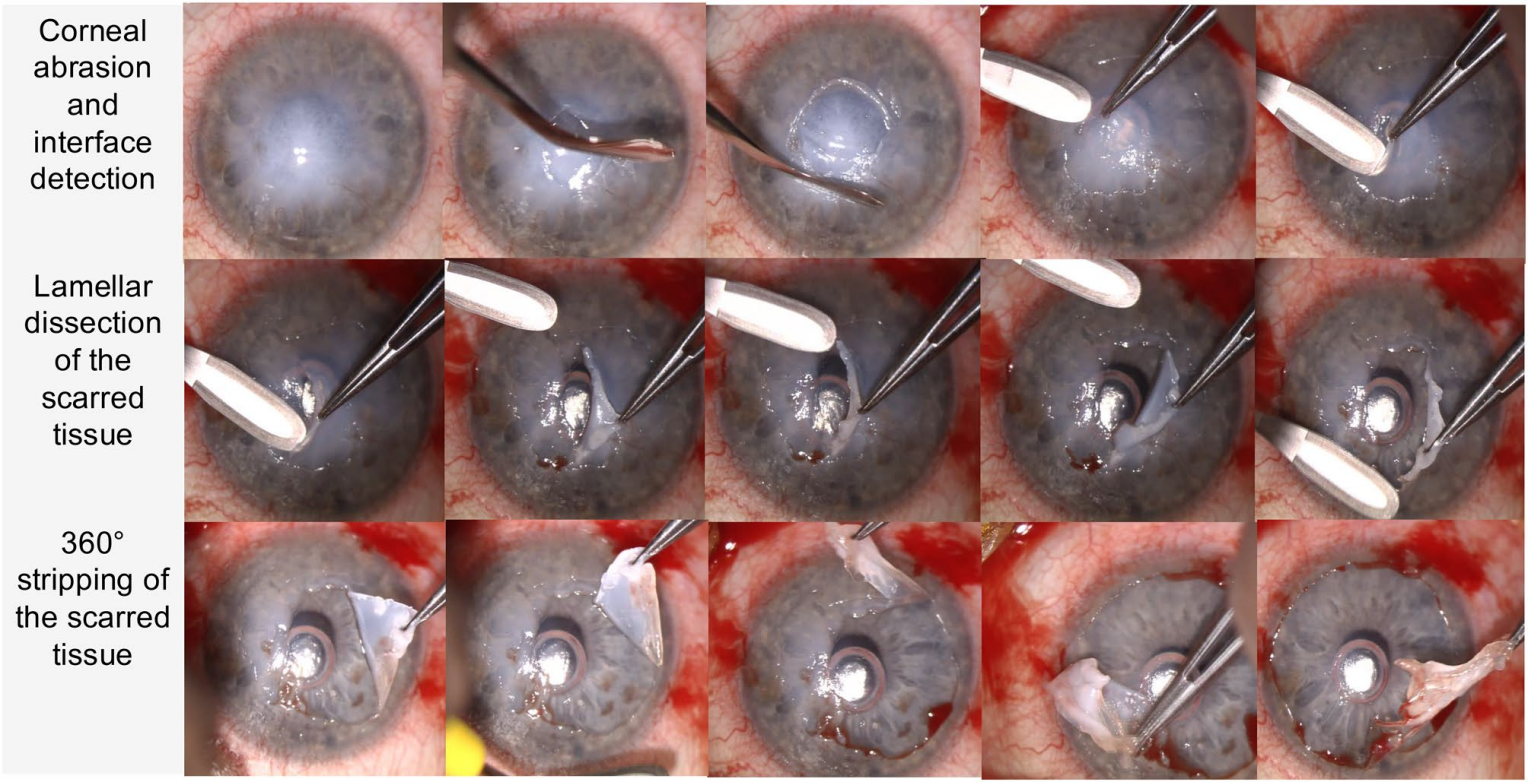
Intraoperative microscope view of the steps of fibrotic tissue removal. Upper row (left to right); the dense corneal opacity is visible. The surgery is commenced with corneal abrasion using a hockey knife, followed by the detection of the interface plane with a crescent blade; middle row (left to right); progressing entire lamellar dissection of the fibrosis using the crescent blade until clear corneal stroma is revealed. Lower row (left to right); the fibrotic tissue is then stripped off over 360° of the entire cornea

The surgery was uneventful. After corneal debridement, the subepithelial tissue was carefully dissected in a lamellar fashion using a crescent knife; the interface was identified relatively quickly. Then the tissue could be readily stripped revealing a relatively clear corneal stroma (Fig. 2). Neither the Bowman layer, nor the anterior stroma appeared to be affected. Bleeding limbal vessels were cautiously cauterized before we proceeded with repeat phakic DMEK followed by amniotic membrane transplantation (overlay) to support corneal re-epithelialization. At the end of the surgery subconjunctival dexamethasone and gentamicin were administered. The histopathological work-up of the excised tissue confirmed subepithelial fibrotic changes.

Postoperative medication included ofloxacin 3 mg/1 ml eye drops four times daily for two weeks, as well as diclofenac 1 mg/1 ml and dexamethasone 1 mg/ml eye drops each four times daily for four weeks. One week after surgery, lubricating eye drops were added hourly and dexamethasone was switched to fluorometholone 1 mg/ml four times daily one month postoperatively. Postoperatively, graft adherence was assessed using AS-OCT.

When the amniotic membrane overlay was removed one week after surgery, the cornea had already cleared significantly. Only some minor superficial corneal haze was visible as were some moderate crystalline lens opacities following previous complicated primary DMEK with postoperative pupillary block. Pachymetry decreased to 545 µm at one week and further to 470 µm at six weeks postoperatively; the DMEK graft was completely attached. BCVA improved to 20/80 (0.25) at one week (after overlay removal) and 20/40 (0.5) six weeks postoperatively. As the patient was satisfied with the early visual outcome, we recommended to await further deswelling before deciding if cataract surgery is required. Corneal neovascularization did not recur during the follow-up period.

Written and signed informed consent for publication was obtained from the patient before manuscript submission.

## Discussion and conclusions

The current case demonstrates that chronic DMEK failure with a dense corneal opacity can be successfully treated with repeat DMEK combined with anterior lamellar keratectomy when the opacity presents as a potentially separable layer on preoperative AS-OCT.

Although long-standing corneal edema is known to cause different degrees of corneal haze and scarring, the severity seen in our case has not previously been explicitly reported to be treated with DMEK [9–11]. Like in our patient, PKP is often recommended to restore visual acuity in such opaque corneas [5]. However, the extent of scarring at which DMEK remains feasible and reasonable, or PKP becomes preferable, remains debated.

To reduce mild to moderate anterior corneal opacifications in eyes with endothelial disease, DMEK was combined with epithelial debridement and mitomycin-C, or was performed after excimer laser keratectomy [10, 11]; deep anterior lamellar keratoplasty was combined with DMEK when stromal scarring was noted [12]. In contrast, the corneal opacity in our case, was considerably thicker and localized between the epithelium and stroma. Consequently, the recipient stroma could be preserved, allowing epithelial debridement followed by lamellar keratectomy and DMEK.

In early FECD, slit-lamp biomicroscopy may be sufficient to distinguish corneal pathologies from healthy tissue. This may, however, be more challenging in advanced disease due to the light scattering in the swollen cornea [13].

Although AS-OCT is commonly used to evaluate post-keratoplasty corneas, the examination is often not routinely performed or evaluated preoperatively. Our reluctance to perform a ´high-risk´ PKP in a relatively young patient (previous FECD) with multiple systemic diseases made us explore alternative strategies. Slit-lamp examination implied that the entire cornea was diffusely scarred, whereas on AS-OCT a compact hyperreflective layer was distinguishable from the less dense epithelium and stroma which was less affected than initially assumed. Consequently, a less invasive surgical approach, namely pannus removal combined with repeat DMEK in a single session, could be pursued [14]. Similarly, in an earlier study AS-OCT could visualize graft detachment in edematous post-DMEK eyes, whereas slit-lamp examination and Scheimpflug imaging were not successful in picturing the delicate DMEK graft [15].

Intraoperative pannus removal was similar to the procedure used for Salzmann nodular degeneration; however, in our case the fibrotic tissue was comparatively thicker, involved the central cornea, and appeared less adherent to the underlying stroma. Had a clear dissection plane not been identified intraoperatively, conversion to PKP would likely have been necessary.

Chronic corneal edema secondary to endothelial dysfunction has been associated with corneal neovascularization, possibly due to impaired epithelial anti-angiogenic function [16]. Regression of neovascularization has been reported following DMEK, which may be related to the restoration of endothelial function and reduced inflammation as could also be seen in our case [17].

In addition, previous studies have demonstrated improved PKP/DMEK survival following regression of corneal neovascularization after cauterization, either alone or combined with topical bevacizumab [6–8]. In our case, cauterization was performed soley to control bleeding from the amputated vessels after pannus removal. As this was the first case of its kind at our clinic, an amniotic membrane transplantation (onlay) was additionally applied to promote epithelial healing and to benefit from its regenerative, anti-angiogenic and anti-inflammatory properties [18]. Six weeks after surgery the entire epithelium was intact, no corneal neovascularization had recurred, and no allograft reaction could be noted, however, longer follow-up results may need to be awaited.

This case highlights that in eyes with endothelial dysfunction and significant corneal opacification, the decision for the appropriate keratoplasty technique may not rely only on the slit-lamp examination. Preoperative AS-OCT may serve as a key imaging tool to determine whether a corneal opacity corresponds to potentially separable subepithelial fibrosis overlying the stroma. Lamellar keratectomy combined with DMEK may provide acceptable outcomes while avoiding PKP, systemic immunosuppression and their associated risks. This insight may be particularly relevant for surgeons who routinely perform full-thickness rather than lamellar corneal transplantations and who do not regularly use preoperative AS-OCT.

Given the short postoperative follow-up period of six weeks, future studies are needed to evaluate long-term outcomes of DMEK and pannus removal in a larger series of similar cases. Additional histological evaluation may further clarify why some eyes develop pronounced corneal fibrosis in endothelial dysfunction whereas others do not.

## Data Availability

Data sharing is not applicable to this article as no datasets were generated or analysed during the current study.

## Abbreviations

AS-OCT: Anterior segment optical coherence tomography
BCVA: Best-corrected visual acuity
CF: Counting fingers
DMEK: Descemet membrane endothelial keratoplasty
FECD: Fuchs endothelial corneal dystrophy
RE: Right eye
LE: Left eye
PKP: Penetrating keratoplasty
